# Are our ‘UHC systems’ learning systems? Piloting an assessment tool and process in six African countries

**DOI:** 10.1186/s12961-018-0340-y

**Published:** 2018-08-06

**Authors:** E. Akhnif, J. A. Kiendrebeogo, A. Idrissi Azouzzi, Z. Adam, C. P. Makoutode, S. Mayaka Manitu, Z. Njoumemi, A. Gamble Kelley, B. Meessen

**Affiliations:** 1 Ministry of Health, Rabat, Morocco; 2 Université Catholique de Louvain, Rabat, Morocco; 3 Institute of Tropical Medicine, Antwerp, Belgium; 4 University Ouaga 1 Professor Joseph Ki-Zerbo, Ouagadougou, Burkina Faso; 5 Ministry of Health, Rabat, Morocco; 6 Regional Public Health Institute Comlan Alfred Quenum, Cotonou, Benin; 7 School of Public Health, Kinshasa, Democratic Republic of the Congo; 8 Health Economics Research and Evaluation for Development Results Group, Yaoundé, Cameroon; 9 Department of Public Health Faculty of Medicine and Biomedical Sciences, University of Yaoundé, Rabat, Morocco; 10 Results for Development, Washington, DC, USA; 11 Community of Practice “Performance Based Financing”, Antwerp, Belgium

**Keywords:** Universal health coverage, Health system, Learning organisation, Participatory research, Capacity-building, Africa

## Abstract

**Background:**

If there is one universal recommendation to countries wanting to make progress towards Universal Health Coverage (UHC), it is to develop the learning capacities that will enable them to ‘find their own way’ – this is especially true for countries struggling with fragmented health financing systems. This paper explores results from a multi-country study whose main aim was to assess the extent to which UHC systems and processes at country level operate as ‘learning systems’.

**Method:**

This study is part of a multi-year action-research project implemented by two communities of practice active in Africa. For this specific investigation, we adapted the concept of the learning organisation to so-called ‘UHC systems’. Our framework organises the assessment around 92 questions divided into blocks, sub-blocks and levels of learning, with a seven scale score in a standardised questionnaire developed during a protocol and methodology workshop attended by all the research teams. The study was implemented in six francophone African countries by national research teams involving researchers and cadres of the ministries involved in the UHC policy. Across the six countries, the questionnaire was administrated to 239 UHC actors. Data were analysed per country, per blocks and sub-blocks, by levels of learning and per question.

**Results:**

The study confirms the feasibility and relevance of adapting the learning organisation framework to UHC systems. All countries scored between 4 and 5 for all the sub-blocks of the learning system. The study and the validation workshops organised in the six countries indicate that the tool is particularly powerful to assess weaknesses within a specific country. However, some remarkable patterns also emerge from the cross-country analysis. Our respondents recognise the leadership developed at governmental level for UHC, but they also report some major weaknesses in the UHC system, especially the absence of a learning agenda and the limited use of data.

**Conclusion:**

Countries will not progress towards UHC without strong learning systems. Our tool has allowed us to document the situation in six countries, create some awareness at country level and initiate a participatory action-oriented process.

## Key messages of the paper

- Countries need more guidance as to how they could develop systemic learning capacities to support Universal Health Coverage (UHC). This requires first that we develop know-how for the measurement of these capacities.
- We have developed a framework and an assessment tool based on the learning organisation concept. The assessment raises sensitive questions. A participatory approach seems particularly appropriate.
- Our pilot application in six African countries indicates that involving UHC actors in the evaluation process enhances the chance of corrective collective actions in the follow-up of the assessment.

## Background

Over the last few years, Universal Health Coverage (UHC) – defined as the capacity to provide all people with access to needed health services of sufficient quality to be effective, while also ensuring that the use of these services does not expose the user to financial hardship [1] – has gained momentum at global level. If the goal is clear, the path to get there is proving not to be easy for many low- and middle-income countries (LMICs).

There is an emerging body of literature on the paths to UHC [1, 2]. For instance, a recent study looked at 24 countries and highlighted the existence of diversity in paths, in strategic choices and, obviously, in results. In fact, for any country, the road to UHC is inextricably linked to the complex process by which policy decision takes place [3]. Thus, the transferability of experiences from one country to another is somewhat limited [3, 4]: "*each country will have to find its own way to reach UHC"*. This statement actually hints at what might be the only generic recommendation to countries, that each must develop its capacity to find its own way to attaining UHC, i.e. to learn.

Over the last decade, global experts have recommended active strategies to strengthen health systems, with several stressing the need for better learning [5, 6]. Health systems are complex systems [7]; under such configurations, deterministic causal models have their limitations and the capacity to learn from emerging phenomena is key [8]. It has also been recommended to adopt new ways of thinking to close the knowledge–action gap; each innovation in health systems should constitute a learning opportunity [9].

However, to our knowledge, no one has thus far provided clear guidance as to how countries could develop learning capacities to support systemic goals such as UHC. One does not even know how to measure these capacities. This gap may have several reasons, including the tendency by some actors to promote solutions for specific priorities instead of strengthening core capacities at system level [9]. As experts from the south or working on southern health systems, we believe that learning capacities at system level should receive much more attention, both by countries and their partners.

Together, we launched a multi-country collaborative project to measure the extent to which what we propose to call the ‘UHC system’ is actually a learning system (LS). By ‘UHC system’ we mean the set of actors and organisations directly involved in the development of the UHC agenda at country level. This set of actors differs from one country to another; but traditionally, it will be comprised of the Ministry of Health, together with other actors such as aid partners, other relevant ministries, private sector, civil society organisations and academia. The concept of LS refers to a translation of the concept of the learning organisation (LO) to a larger system. It incorporates both the dynamic between its constituting organisations and the interactions that happen within each organisation.

So far, few researchers adopted the lenses of LSs to look at the health sector. However, interest is growing, especially in high-income countries. For instance, Friedman et al. [10] proposed the following attributes of a learning health system (LHS): the LHS is (1) trusted and valued by all stakeholders, (2) economically sustainable and governable, (3) adaptable, self-improving, stable, certifiable and responsive, and (4) LHS capable of engendering a virtuous cycle of health improvement. In another work, Rubin et al. put forward a nice metaphor: *“The LHS can be seen as the tapestry that emerges from weaving together efforts across the health information management, health IT, patient engagement, clinical care, research, and public health arenas aimed at utilizing data, information, and knowledge to improve health*” [11]. This paper deals with this issue of LHS in low-income countries, especially in French-speaking Africa. It presents the history of the project, underlying concepts, the framework, our tool to assess whether a country’s ‘UHC system’ is a LS, its implementation and our findings. The tool was structured in a way to assess the extent to which learning capacities and processes are in place, whether the environment is conducive for learning and leadership promotes learning. In this research, we focused on the types of learning that are related to the UHC actions. We use our six countries study as an opportunity to validate our tool and the implementation process.

In the next sections, we first present how we have adapted the concept of LO for an application to audit ‘country UHC systems’. We then share insights collected in the six countries where the research was carried out. We identify areas of weaknesses that impede progress towards a LS. We conclude the paper with a general reflection on the approach and the instrument we have developed and provide guidance for further work in this area.

Since 2009, the multi-agency platform Harmonisation for Health in Africa has supported several communities of practice (CoPs) – each CoP is made up of experts committed to advancing, through exchange and co-production, thematic knowledge identified as key for better performing health systems in Africa [12, 13]. A strength of these CoPs, beside their size (most of them have more than 1000 expert members), is that they bring together experts working at different levels of the knowledge–policy chain, at country, regional and international levels [14].

In 2013, experts from two CoPs (Financial Access to Health Services and Performance-Based Financing) agreed that the fragmentation of healthcare financing was a major problem in their countries and a real challenge for progressing towards UHC. The CoPs developed a collaborative research project (sponsor: UNICEF/*Fonds Français Muskoka*). The first phase of the collective work documented the reality of this fragmentation in 11 countries (23 schemes on average per country) [15]. The documentation process also revealed the high fragmentation and inadequacy of information at national level – clearly, a key bottleneck for any steward attempting to bring order to the existing patchwork of financing schemes to expand UHC.

The research team henceforth decided that the next phase of the project should focus on assessing the capacity of each country to handle this complexity, and more particularly the learning capacities of what researchers decided to call the ‘country UHC systems’, i.e. the group of key organisations and stakeholders involved in UHC dynamics and implementation, with the recognition that some organisations have a central role and others a more peripheral one.

After a rapid examination of the literature, we found that the concept of LO could be a powerful way to approach how countries manage knowledge to progress towards UHC. In business and organisational studies, there is today a vast literature on LO. The concept has been developed to acknowledge that learning is key for any organisation to thrive or even survive in a fast changing and competitive environment. There are various definitions of a LO [16–19]; in a nutshell, a LO is an organisation continuously using the three key steps of any learning process, namely (1) intelligent collection of new information, (2) combination of the new information with its pre-existing stock of knowledge and interpretation, and (3) conversion of the new enriched knowledge into action.

To inform our research, the first author of this paper carried out a scoping review with a focus on the applications of LO to the health sector. It revealed that the concept of LO was receiving growing attention in the health sector, yet there were few applications to LMICs [20]. The review also showed that most of the applications were at the level of hospitals and health centres, with only two applications of the LO concept to health systems and none to UHC. We thus had to develop our own approach, including designing a new research instrument. The review gave us an overview on possible frameworks and research methods. Most of the LO frameworks converge towards the models of Senge [16] and Garvin [17]. One of the main findings from the review was very consistent with our own long-term objective, namely that managers of the health organisations that have used the LO frameworks found it an important added value to improve the overall performance of their organisation by linking the learning to the action and by creating a learning dynamic.

## Methods

In this section, we report how we applied the LO concept to the ‘UHC system’.

### Design stage

The process of the study was collaborative and participatory from the beginning. Through the online platforms of the CoPs, the research coordinators (BM, AGK and HEA) invited countries to apply to participate in the multi-country research and to meet two conditions, namely to have a mixed team composition (one researcher, one cadre of the Ministry of Health and one cadre of another ministry involved in UHC) and have an official backing at the ministerial level. Delegations from 11 countries successfully applied and were invited to a launching meeting in Rabat, Morocco, in order to contribute to the development of the protocol and methodology, including the data collection tool. Together, we agreed that the main objective of the research would be to audit the ‘UHC systems’ to assess to what extent they function as LSs. By applying the tool in several countries, it was expected that benchmarking across participating countries would allow us to identify areas for which there could be cross-country learning (i.e. areas for which one country performs better than others) at a later stage, but also possible areas for regional intervention (i.e. areas in which most countries perform poorly). These were two actions for which the CoPs could play a constructive role.

Our hypothesis is that, in order to progress towards UHC in a specific country, actors and organisations involved in the UHC agenda must operate as a coordinated and LS in order to adopt and adapt effective strategies to achieve the UHC goal. The first output of the workshop was therefore a common strategy for mapping actors and organisations of the national ‘UHC system’. They would be the organisations under scrutiny and covered by the sampling of key informants. We agreed that the actual composition of a ‘UHC system’ was partly country specific and that its boundaries were fuzzy.

**Fig. 1:**
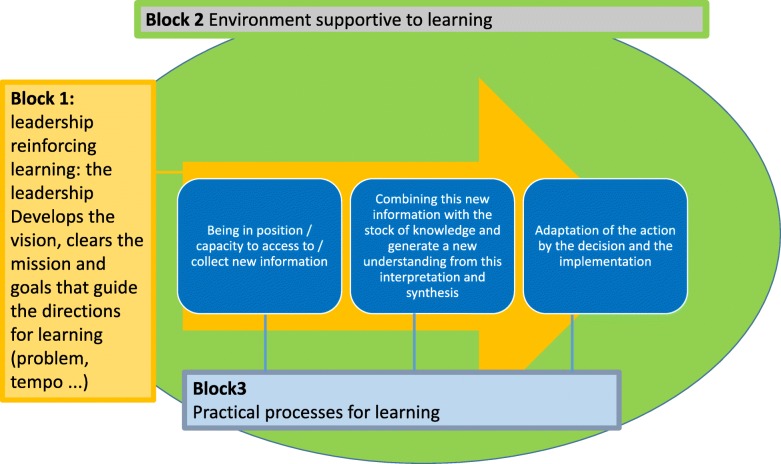
Conceptual framework of a learning system

The second output of the workshop was our framework and the questionnaire itself. Among the different existing LO frameworks [21–29], we opted for the one proposed by Garvin et al. [28]. We found it comprehensive, well organised and adaptable to the ‘UHC system’. This decision was consistent with our literature review [20], indicating that Garvin’s LO framework was indeed one of the most commonly used in health sector studies. With workshop participants, we adapted the original version of the framework to take into account the characteristics of a ‘UHC system’ and the specific context of LMICs. Our final version of the framework is organised around Garvin’s three main blocks of (1) leadership reinforcing learning; (2) environment supportive to learning; and (3) practical processes for learning (for our own graphic representation of the framework) (Fig. 1).

We have opted for a research design already validated by other LO empirical studies [20], involving the cross-sectional administration of a standard questionnaire tool to persons who are familiar with the case under investigation (for applications to health centres or hospitals see, for instance, Kelly et al. [30], Leufvén et al. [27], Mohebbifar et al. [31]). Our own survey tool is inspired from the tool developed by Garvin et al. [28]. At our workshop, for each of the three blocks, participants were tasked to develop questions depicting an attribute one can expect from the ‘UHC system’ if it is in a learning mode. A few weeks later, the research team finalised a list of 92 statements on which key informants were requested to give their opinion (the survey tool is available on request from the last author).

Under block 1, 20 statements measure whether leadership by authorities is supportive to learning for UHC. Under block 2 (supportive environment), six statements assess the autonomy of individuals and teams (sub-block 2.1), six relate to the ability to integrate required expertise (sub-block 2.2.), seven measure the collaborative culture (sub-block 2.3), five deal with the openness to new ideas (sub-block 2.4) and seven assess whether there is the technical culture required to develop UHC (sub-block 2.5). Under block 3 (processes enhancing learning), our instrument checks whether the ‘UHC system’ has a learning agenda (sub-block 3.1, eight statements), whether there is room for experimentation block 3.2, five statements), whether experience is valued (sub-block 3.3, eight statements), how intelligence is organised (sub-block 3.4, 16 statements) and whether learning is translated into action (sub-block 3.5, four statements).

Our tool thus tries to capture the multidimensional reality of learning for UHC, by acknowledging the various types of relevant knowledge, the distribution of roles within the community of actors, the efforts to be done by some key players, the capacity issues, the importance of dedicated processes and resources, including platforms bringing different knowledge holders around a common learning agenda.

Learning can occur at four levels – individual, team, organisation or system. To each question, we attributed one of these levels. We added the system level to the initial definitions we found from the literature to take into account the specificities of the UHC system [21].

Similarly to Garvin et al. [28], we used a 7-level scale for each question; the interpretation of each score is given as follows: 1 = I find this statement highly inaccurate, 2 = moderately inaccurate, 3 = slightly inaccurate, 4 = hesitate between accurate and inaccurate, 5 = slightly accurate, 6 = moderately exact, 7 = strongly exact. There was also the possibility to express no opinion.

### Implementation

The first task assigned to country research teams was to map their ‘UHC system’ by identifying organisations with an important role in the UHC agenda – this was rather easy as many countries were busy with this agenda, sometimes with established multi-actor bodies (e.g. steering committee, taskforce) (for country lists, see country reports available at request).

As for the administration of the survey tool, it was requested to select at least 30 persons from the organisations and actors mapped as part of the ‘UHC system’. Research teams were encouraged to target staff members playing a key role in the systemic efforts for UHC (e.g. persons in coordination bodies). We set as a rule that there should also be persons from the decentralised level among the informants. Organisations with a dominant role in the country ‘UHC system’ (e.g. Ministry of Health) should be more represented than those with a minor role. In accordance with ethical requirements, it was agreed with the research teams that the administration of the questionnaire should ensure the anonymity of the respondent as well as the confidentiality of the collected information.

While we had a small core funding for the coordination of the research, resources for country work were very limited. Each team was requested to raise funds for its work at country level – this was also seen as a strategy to increase buy-in by the country ‘UHC system’. From the 11 teams present in the launching workshop, six were actually able to implement the research. Among these six country teams, a principal investigator was identified; they acted as a focal point in the interaction with the coordination team based at the Institute of Tropical Medicine and the five other teams. Throughout the whole development and implementation process, exchanges between principal investigators facilitated learning across teams.

The research protocol was approved by the Institute of Tropical Medicine institutional review board (number 996/15). Ethical clearance was also obtained at country level when it was required. The study started in June 2015 and data collection was finalised in early 2016. A last international meeting was organised in January 2016, where we shared our respective findings and devised a standardised analysis approach to adopt for the national reports of the study.

The findings presented in this article are extracted from the database, but also, for the qualitative part, from the reports produced by the six country teams. For three of the six countries, the report was enriched by national validation workshops, which deepened our understanding of the learning capacity of the UHC system. In these workshops, country UHC system actors were invited to participate, including most of those interviewed.

## Results

First, we present the principal findings from each country according to the main elements of the framework, and then we conduct a cross-country comparison according to the sub-blocks of the framework. We collected a sample of responses from 239 interviewees (in alphabetical order: 31 for Benin, 40 for Burkina Faso, 45 for Cameroon, 43 for Democratic Republic of the Congo, 40 for Morocco and 40 for Togo). For Benin, the size is smaller than all other countries because of the presidential elections; yet, the main organisations of the UHC system were represented.

We have calculated and analysed average scores and their variations for each country and sub-block of the framework (Table 1) and the average by level of learning (individual, team, organisation and system) (Table 2).

**Table 1:** The scores and their variation by country and by sub-block

	Benin		Burkina Faso		Cameroon		Democratic Republic of the Congo		Morocco		Togo	
	Mean	SD	Mean	SD	Mean	SD	Mean	SD	Mean	SD	Mean	SD
1.1. Leadership that reinforces learning	4.9	1.4	5	1	4.7	1.4	4.9	1.2	5.1	1.2	4.9	1.1
2.1. The autonomy of individuals and teams	5	1.7	5	1.4	4.9	1.5	5	1.4	4.8	1.4	5	1.1
2.2. Ability to integrate the necessary and appropriate expertise	5.4	1.2	5.2	1.3	5.1	1.4	5.3	1.2	5.3	1	5.1	1.1
2.3. Collaborative culture	5.2	1.2	5.3	1.1	4.7	1.4	5.1	1.1	5	1.4	5.2	1.2
2.4. Openness to knowledge and ideas held by individuals	4.9	1.7	5.4	1.1	4.8	1.3	5.1	1.2	4.7	1.4	4.9	1.2
2.5. Technical culture required to develop UHC	4.6	1.5	5	1.2	4.2	1.4	4.8	1.3	5	1.3	4.6	1.3
3.1. Learning agenda	4.9	1.3	4.5	1.3	4.1	1.5	4.1	1.3	4.1	1.5	4.2	1.5
3.2. Experimentation	4.5	1.7	5.4	1	4.5	1.6	4.7	1.5	4.9	1.3	4.7	1.5
3.3. Experience	4.9	1.4	4.9	1.3	4.3	1.4	4.8	1.3	4.6	1.3	4.8	1.4
3.4. Intelligence and expertise	5	1.3	5.1	1.1	4.6	1.3	5.1	1.1	5	1.1	4.7	1.4
3.5. Synthesis and action	5	1.8	4.9	1.4	4.5	1.6	5	1.1	4	1.5	4.7	1.5

**Table 2:** The scores and their variation by levels of learning

		Benin	Burkina Faso	Cameroon	Democratic Republic of the Congo	Morocco	Togo
Individual level	Mean	4.7	5.3	4.8	5.5	4.8	4.9
	SD	1.5	1.1	1.3	1.2	1.6	1.3
Team level	Mean	4.9	4.9	4.7	5	4.4	4.9
	SD	1.5	1.2	1.4	1.3	1.3	1.1
Organisation level	Mean	4.9	5	4.6	5.1	4.9	4.8
	SD	1.4	1.1	1.3	1.2	1.1	1.2
System level	Mean	4.9	5	4.6	4.5	5	4.8
	SD	1.3	0.9	1.2	1.1	1	1

We will first analyse these findings country per country and will then carry out some cross-country comparisons.

### Analysis for Benin

The data collection in Benin took place during a national electoral campaign. This complicated the interaction with the identified key informants. Although the size of the sample was relatively small as compared to the other five countries, the dynamic of the study was interesting. In general, most of the scores were above 4.5 (Table 1). The validation workshop allowed UHC actors to air several major frustrations with the UHC dynamic in the country under the previous government (a period marked by the failure to roll out a promised Universal Health Insurance). Among other things, they pointed at the poor coordination between ministries (especially on communicating about UHC) and the insufficient involvement of national technical experts, with several key positions entrusted to persons with political profiles. The scores were evaluated as reflecting the reality in the country and sometimes even too positive (participants suggested that the fact that the survey was administrated during the electoral campaign period probably biased scores upward for a few sensitive questions). Participants mentioned that, in their country, information is a source of power, which can thus hinder the sharing and the spreading of knowledge at all levels of the UHC system. Participants listed some possible actions to strengthening the learning capacity of the UHC system, including (1) the creation of a national community of practice platform to share knowledge; (2) some training on leadership for leaders of UHC organisations; (3) the creation of a group of experts to promote the LS culture; and (4) auditing the existing strategies to identify bottlenecks and suggest corrective actions. The ‘Hub Benin’ is in place and its online forum is already an active place of discussion about UHC.

### Analysis for Burkina Faso

Our study indicates that among the six countries, Burkina Faso was the most advanced in establishing a LS for UHC. Indeed, the average score hovered around the level of 5 (Table 1), often with a small standard deviation – an indication that there was a high consensus among respondents. Participants of the validation workshop confirmed this general picture. They identified some of the specific strengths of their ‘UHC system’, as follows: (1) The creation of a sectoral framework for dialogue on health and nutrition (*cadre sectoriel de dialogue santé et nutrition*) under the leadership of the Ministry of Health. This is a platform for broad exchanges between actors on major issues of the health sector. It somehow is fostering knowledge sharing and strengthening the coordination for the ‘UHC system’. (2) The relative high number of health research institutes (at least five in the public sector) that produce considerable scientific knowledge, and in the recent past significant research in the field of UHC. (3) The existence of highly qualified executives within ministries and departments involved in UHC. This has led to the emergence of a whole supportive ecosystem.

The study and workshop also gave UHC actors an opportunity to identify areas of weaknesses. This was mainly based on a review of questions with relatively lower scores (15 statements). Among other things, participants of the workshop agreed that the coordination among UHC actors could be improved and a better use of knowledge by the lower levels of the system is required to get concrete results. Seven of the 15 questions that had low scores were related to the lack of a UHC learning agenda and to the capacity to integrate the right expertise and scientific knowledge into the system. Inspired by our assessment, participants proposed the following elements of action: among others, developing a strategy for knowledge management in the system, creating a platform for sharing knowledge, giving more autonomy to teams and individuals in the public administration, and better involving research organisations in the development and implementation of policies.

### Analysis for Cameroon

In Cameroon as well, the study received high-level support from the National Task Force Group on UHC and the Ministry of Health. The discussion of the results in a meeting involving a representation of almost all UHC actors confirmed that the levels of the scores for each block and sub-block reflect the reality in the country. The standard deviation varied between 1.3 and 1.6, indicating a good level of consensus among interviewees. The study highlighted some strengths in the UHC system in Cameroon, especially regarding the capacity to integrate the necessary expertise into the system, the autonomy of individuals and teams, and the existence of a collaborative culture (Table 1). This might be catalysed by the existing political willingness to move towards UHC, as evidenced by the creation of a National Task Force Group for UHC. The study put the spotlight on areas of weaknesses such as (1) learning agenda, (2) synthesis and action, and (3) the process of sharing experiences among individuals. The research team, on the basis of discussions which occurred during the validation workshop, proposed a list of concrete actions to further move the UHC system towards a LS. These included (1) the creation of a platform for exchange and sharing that involves all actors, including the civil society, (2) the development of policy briefs to promote a LS culture, and (3) capacity-building and the development of a learning agenda. It is important to mention that, despite the novelty of the LS concept, participants in the validation workshop showed interest as to the use of the tool for diagnosing and evaluating actions aiming at developing a UHC LS. In October 2016, the exchange platform was set up (the ‘Hub Cameroon’).

### Analysis for the Democratic Republic of the Congo

In the Democratic Republic of the Congo, analysis by main blocks showed that the UHC system has quite good scores (around 5) for the blocks related to leadership and a supportive learning environment (block 1 and 2). The analysis of scores by levels of learning (individual, team, organisation and system) provided an interesting pattern, wherein the highest scores were found for learning at the individual level (5.47) while learning at system level had the lowest score (4.5) (Table 2). This observation led us to speculate that, in loosely regulated countries like the Democratic Republic of the Congo, there could be a trade-off between these two levels of learning, in the sense that experts are able to seize many opportunities for individual learning (thanks to aid projects) but without a benefit or even to the detriment of the whole system. The capacity to integrate new expertise in the UHC system scored high (5.28), which indicate the openness of the system to use the expertise in its environment.

The study also highlighted some areas of weaknesses (Table 1); for instance, the learning agenda (sub-block 3.1) and experimentation (sub-block 3.2) scored lower than the others. It provided a diagnostic of the level of development of the LS, which could be used in future projects.

### Analysis for Morocco

Our study in Morocco benefited from particular momentum. The Secretary General of the Ministry of Health was very aware of the need to improve the learning capacities of the national health system. This study was actually a major source of inspiration for a new project developed by the Ministry focusing on knowledge management for health system strengthening. It has indeed provided areas of strength and weaknesses that could further be used to plan actions to develop a LS.

Several weaknesses were identified (Table 1), notably around (1) the learning agenda, (2) synthesis and action, and (3) the development of teamwork. The results of the study showed a good score for the leadership level. A primary explanation of the good score for the leadership block (Table 1) is the high level of involvement of the government in UHC issues. Indeed, the creation of a high interdepartmental committee chaired by the head of government has strengthened the leadership for learning because of the generated need to prepare the meetings of this committee. One of the reasons for the low score for the ‘synthesis and action’ sub-block is the limited development of strategic purchasing in Morocco. This ‘backwardness’ was confirmed at a regional workshop organised by WHO and the same CoPs in Rabat, in late September 2016.

The national team has already identified actions to be considered to move towards LS, mainly creating a platform for sharing knowledge by using the latest technology, broaden the occasions and spaces for meetings to share knowledge among actors, and better position research to ensure more use of the synthesised knowledge in the policy-making process.

### Analysis for Togo

The study demonstrated the relevance of our tool to portray to what extent the UHC system in this country performs as a LS. The validation workshop confirmed the scores for each block and sub-block with a high level of consensus evidenced by the low value of standard deviations. The scores showed some strengths in terms of existing attributes of a LS, but also many areas where much progress still needs to be made. Globally, the study showed that the blocks of leadership and supportive learning environment scored around 5 (Table 1), which we deem puts it above the threshold where one begins to see the attributes of a LS (4.5). This relatively higher score might be explained by several system-level efforts such as improvements in the information system and awareness about the necessity to improve the coordination and the governance of the UHC system. The study also highlighted areas of weaknesses, especially for the ‘learning agenda’ and the ‘synthesis and action’ sub-blocks, which could result from the existence of parallel but poorly coordinated information systems and the lack of a clear agenda for knowledge sharing. The national validation workshop facilitated an in-depth analysis of results and a discussion of an action plan for the next phase of the study. These actions mainly focus on (1) the creation of a platform for knowledge sharing (set up early 2017), (2) the capacity-building of all actors involved in the UHC process, and (3) the development of a roadmap to further move towards a UHC LS.

### The comparison among the six countries

Our study does allow comparison of the situation across the six countries, but with several caveats. First, the instrument was implemented by different research teams in the six countries, sometimes with variance in terms of administration techniques and, second, responses may be culturally biased – one could imagine that some societies are more open to self-criticism than others. Still, the comparison is interesting; it indicates that the UHC systems have shortcomings, some common across the six countries.

To test the sensitivity of our questions we first analysed the frequencies of the score ‘0’ (answer ‘no opinion’) across our 92 statements. Our hypothesis is that, if a question has many 0 scores across countries, this may indicate a poor formulation or inappropriate question. This seems to be the case with question 57 (The state budget takes into account the learning for the UHC system), where the total number of 0 scores in the 239 questionnaires collected was 53*.* If one observes more 0 scores in a country than in another, this may indicate that the respondents received less information to understand its meaning or that the respondents were less comfortable with answering this question (or not knowledgeable enough). For instance, it is in Benin that respondents provided the greatest number of 0 answers for block 1 (leadership) – this may be due to the fact that the surveys were administrated during an electoral period. For further analyses on average scores, all the 0 scores were removed. For the 92 questions, the highest average number of questions with a 0 score was observed in Benin (11 out of 92 per interviewee on average), while the lowest average was observed in the Democratic Republic of the Congo (4 out of 92 per interviewee on average).

In this paper, we propose to focus our comparison mainly on the sub-blocks of the framework, which give a clear vision about how the attributes of a LS are developed in a given country. When relevant, we also report on item questions, as they capture very specific issues.

In general, all average scores were above 4 and most of them above 4.5 (Table 1). The vast majority of respondents in each country assessed their UHC systems positively overall, which can be interpreted as a constructive view on the emergence of learning capacities in their system.

Some sub-blocks scored better across countries. The best one was ‘ability to integrate the required expertise’ (sub-block 2.2), under which one can find some of the highest scores from the 239 respondents. Some examples are question 30 (The analytical contributions from technical and financial partners are useful for informing progress towards the UHC) (average: 5.96) and question 28 (In the absence of in-house expertise, subcontracting of expertise is possible on own funds or on the resources of technical and financial partners) (average: 5.51). It also seems that respondents were quite satisfied with the collaborative culture (sub-block 2.3) – this was particularly true at the organisation level. This sub-block received a particularly high score, varying from 5 for Cameroon to 5.57 for Benin (organisational level varies from 4.95 for Cameroon to 6.09 for Morocco with an average across countries of 5.47).

There were also sub-blocks and items that were weak across countries – such a finding is particularly interesting because it helps clarify priority areas for regional action. This is certainly the case for sub-block 3.1 related to the learning agenda, wherein most countries have a score of around 4. Three of the five questions with the lowest score of the whole questionnaire actually belong to this sub-block.

Some cross-cutting and interesting patterns were also observable at the question level. While question 1 (on the importance of coordination across ministries and actors for succeeding UHC) ranked second in terms of score (average score on 239 respondents: 5.74), the fifth worst score related to the coordination among the same actors in terms of communicating the UHC agenda to the general public (average score on 239 respondents: 4.1). Therefore, it seems that today, in Francophone Africa, there is a strong dynamic of organising actors around the UHC agenda, but the group as a whole communicates in a very confused manner externally.

Our survey also revealed a real structural problem with the triad ‘technology, data and decision making’. From the whole set of 92 questions, the question with the lowest score (3.63) was the 80th (In our UHC system, every player, even at decentralised level, has access to up-to-date data and to an analytical interface enabling them to assess their own performance). Interestingly enough, this answer retrospectively validates the focus of a workshop recently organised by another CoP on health systems delivery [32] – a reassuring sign of the aptitude of this other CoP to identify and address real problems. In reality, the problem with data is broader; the scientific culture of using quantitative data (the 45th question) got low scores as well, with a minimum average score of 4.15 for Cameroon and a maximum average score of 4.84 for Burkina Faso (average on the 239 questionnaires: 4.5). According to respondents, there is also an issue at the level of proactive integration of innovative information and communication technology (46th question, minimum: 4.46 in Togo; maximum: 5 in the Democratic Republic of the Congo; average on the 239 questionnaires: 4.61). Similarly, respondents deem that mechanisms for rapidly integrating information about health facility performance into action (such as strategic purchasing) are under-developed, with a minimum average score of 3.12 for Morocco and a high score of 4.89 in the Democratic Republic of the Congo (which is rolling out Performance-Based Financing as a national strategy).

Our instrument also identified sub-blocks or areas for which there is variation across the six countries. Again, this is interesting, as it indicates areas for possible cross-country learning, for instance, through a joint learning network. Within our sample, Burkina Faso was the most advanced country in terms of the development of its ‘UHC system’, surpassed by another country only for three sub-blocks (Table 1).

Each country can also identify blocks or sub-blocks in which it lags behind the others. Cameroon seems to have a general problem with all of block 3 (practical processes for learning), with its score being indeed very low (4.4) as compared to other countries. One could also identify, for each sub-block, pairs of countries with the largest spread. For instance, if Cameroon wants to improve its leadership for UHC, Morocco would be the country to visit; if Togo and the Democratic Republic of the Congo want to learn how to systematically learn from experimentation, Burkina Faso is the country to visit.

The analysis by level of learning (Table 2) is crucial to identify areas of weaknesses of a system in order to be a learning one. For the individual level, the scores range from 4.7 (Benin) to 5.5 (Democratic Republic of the Congo); this score is more than five for four countries. For the team level, the scores range from 4.4 (Morocco) to 5.0 (Democratic Republic of the Congo), with a value above 5.0 for five countries. The organisational level was identified with scores varying from 4.6 (Cameroon) to 5.1 (Democratic Republic of the Congo), with a value above 5 for the remaining countries. For the system level, the scores ranged from 4.5 (Democratic Republic of the Congo) to 5.0 (Burkina Faso and Morocco). We notice that there is room for developing learning capacities at the four different levels of the system. Indeed, there is no country that scored high in all levels at the same time. Through our participation in the process we have made some observations. Some countries have specific dynamics and the way the learning is developed and shared could be attributable to the organisational culture and the strategies put in place. These learning strategies could be shared among countries.

## Discussion

We pursued two objectives with this participatory study, namely (1) to adapt a framework to assess the extent to which a ‘UHC system’ has the attributes of a learning system and (2) to test this framework in a sample of countries after translating it into a tool to audit ‘UHC systems’ as LSs.

After its completion, we are confident that we have progressed on both objectives. We have been able to generate an informative snapshot of the status of UHC systems in the six countries under investigation. The workshops organised to analyse the results of the study at national levels confirmed the potential of our tool to increase the awareness of decision-makers about the importance of systemic learning for the UHC agenda. The whole process of the research also triggered a rich learning across our countries.

Each country could be singled out for some smart ideas on how to advance learning for UHC. Let us just flag a few. In Morocco, the creation of an inter-ministerial steering committee for UHC allowed a strategic positioning of the UHC agenda at the level of the government, with a clear benefit in reducing the gap between knowledge and strategic decision-making. Burkina Faso shows that even low-income countries can build strong learning ecosystems. Constant investment in health system research capacity pays off.

During our data collection, we realised the heuristic power of our framework, wherein the process revealed to some actors the different elements needed for a leadership supportive to systemic learning. The national workshops encouraged countries to take some concrete actions (with eventually, variable success), for instance, by setting up knowledge platforms. Country reports were rich in recommendations.

The collective analysis of the study results in an international meeting showed that each country has something to share with others in terms of successes but also weaknesses. The dynamic we created throughout this research has triggered an exchange between countries as to how to better learn to achieve the UHC objectives.

The study may contribute to international research on UHC in two ways. First, to the best of our knowledge, this is the first attempt to measure the extent to which countries have systemic learning capacities for UHC. Our framework tries to embrace a comprehensive view of the attributes that matter for systemic learning, including how knowledge can be converted into action and how actions can feed the collective memory of the system knowledge. The comparison within countries, and to a lesser extent across countries, confirms the merits of our tool. Second, we have adopted an original approach that has maximised participation across and within countries. We involved national UHC actors systematically and deeply, from the identification of the broad research question of the overall project to data analyses and interpretation through the validation workshops. With this project, we have ourselves practiced what we recommend, that is to implement activities in such a way that they consolidate the autonomous learning capacity of the national UHC system.

During the implementation of the study, we observed how the tool helped our informants to think about the importance of learning for UHC. At country validation workshops, we were particularly impressed by the capacity of the tool to orient discussions (especially when they are organised around high and low scores among the 92 statements).

Still, our methodological approach can be improved. Many respondents complained about the length of the questionnaire. An effort to reduce the number of questions based on a careful analysis of these six country surveys may then be needed. Some new guidance could also be provided for the administration of the survey. The approach adopted in Burkina Faso – to first invite all the respondents to a 1-day workshop to explain the key concepts (including Universal Health Coverage, which may be understood very differently across actors), the framework (for all our respondents, the LO was a new perspective) and review each question (to avoid misunderstandings) – seems particularly interesting. It could also be relevant to reduce the degree of anonymity. Our choice to protect respondents was a real constraint at the analytical stage; for instance, we were not able to compare opinions from Ministry of Health staff versus those affiliated with other organisations.

In terms of causality analysis, we think that it would be interesting to apply the instrument in countries known to have made good progress towards UHC as a contrast to others performing less well. This could be a way to establish a stronger association between the LS and the UHC objective and to improve the validity of the whole instrument.

These questions related to the validation of the evaluation tool are important; still, according to us, these should not be at the cost of the process itself. Rather, the overall objective of the exercise should be to increase the commitment of UHC actors to learning. So new adaptations (shorter list of questions, simplification of the scoring system, administration through an online survey) should be assessed on this basis.

We must indeed keep in mind the inherent limits of the methodology. A first one is the elusive nature of our concept of ‘UHC system’. Our main concern was to embrace actors beyond the health sector – this is key for this agenda; but for future work, it could be interesting to come with a stricter definition, maybe established on some key functionalities identified as central to the UHC agenda. For example, the ‘UHC system’ could be identified as the set of actors who take an active role in the collective deliberation and action to improve the resource mobilisation, pooling of risks, purchasing and delivery of health services to the national population. A second limit is that our measurement tool gathers opinions, not facts. This approach, which is practiced for other cross-country benchmarking (see, for instance, the corruption perception index produced by Transparency International), is subject to the information held by the respondents and their critical perspective. It is possible that some informants are simply unaware of some strengths of their ‘UHC system’ or, conversely, over-estimate the presence of some processes or practices. To get a more comprehensive picture, an interesting option would be to collect some factual information and to carry out some case studies (for instance, a review of significant situations where learning occurred or did not occur). We have not found a clear method on how to handle the possible fact that some respondents may be more critical in one country than in another.

Our samples were small – the main reason is that the number of experts directly involved in the UHC agenda is never large at country level. We have not tried to calculate confidence intervals; much more than ‘statistical power’, our approach is to build on empowerment through participation. We believe that this action-research approach is legitimate, but our readers must keep in mind its obvious limits. Taking, for instance, just the measurement part of our approach without integrating the participatory and interventional components would seriously reduce its value.

A key feature of our tool is indeed its capacity to identify areas for action. For instance, across countries, we noticed weaknesses in the sub-block ‘learning agenda’. This is not surprising since this has not been a priority recommendation of the international community so far. Today, at country level, learning on UHC is very fragmented. In our view, a national learning agenda on UHC would be beneficial to countries as it would organise actors around the UHC objective. In countries with a stronger knowledge ecosystem (e.g. Burkina Faso), the main issue will probably be to coordinate the many actors. In countries where the scientific ecosystem is not completely in place (e.g. Togo), such a learning agenda would help prioritise the very limited qualified resources. In a country like Morocco, where there are capacities but where attention to UHC is not developed, the learning agenda could be a tool to convince researchers to focus more on UHC.

As reported in our results section, it also seems that our tool (and our study more generally) may contribute to setting momentum around learning at the country level. Indeed, in the six countries, the study was a much-needed first step in a long journey to move towards UHC. In this respect, only time will tell if our work had an impact. The study showed that there are many things to improve in our six countries. After awareness raising, we will have to set up a structured approach to learning at country level. For this endeavour, we can refer to our building blocks, value learning through exchanges between countries (as we already do with our communities of practice), and dare to identify, through comparisons, strengths and weaknesses of various practices.

We believe that our study also sets out an interesting direction for other countries. Progress towards UHC will require that national actors work together and develop enough collective intelligence to ensure that strategic decisions are grounded in knowledge. This vision implies a new culture and other ways of organising health systems. The ambition should be to create environments, practical processes and a leadership supportive to systemic learning.

Beyond the case of the six countries, our work, thus provides a generic roadmap to work on the key elements and conditions for a ‘UHC system’ to become a LS. Indeed, behind each block and sub-block of the conceptual framework, there can be a series of actions to take to progress towards a LS. Decision-makers can check if the ingredients needed to reinforce systemic learning are in their systems; our study also suggests them to explore how other countries have done to consolidate learning capacities in their UHC systems. This research also draws attention to the fact that the journey towards UHC will not just be an endeavour of building explicit knowledge: data analysis and evidence are key for a LS, but learning is much more than that. In fact, it is important that system actors create practical processes for a learning cycle that stretches from the production of knowledge to its storage, sharing and use in action and problem solving. Our hope is that this first work will encourage more countries to audit the learning capacities at the level of their health system. The dynamics initiated through this small network of six countries may inspire other groups. We do not doubt that this agenda will keep many actors busy for the next decade.

Finally, our research illustrates that some of the challenges recurrently discussed within the global health system research community (such a better involvement of decision-makers throughout the research process and a greater attention to implementation issues [33]) can be addressed. We believe it is mostly a matter of openness, innovation and commitment. As CoPs experts, we strongly believe that participatory approaches [34] is the way to go, if one wants to consolidate a LS culture.

## Conclusion

Our ambition was to test and validate a tool to help countries to assess whether their ‘UHC system’ is progressing towards a LS, and to identify areas and actions for improvement. We believe that this is a new field for operational research, especially for LMICs. We hope that our findings will stimulate other teams to conduct similar studies in other contexts.

The journey to UHC will be a long one. This pilot study does not provide direct answers or recipes to develop a LS, but it has kicked off a reflection, at regional and country level, and also suggests future directions for research and action. By its participatory nature from the outset, the large involvement of key stakeholders for the interviews and the organisation of validation workshops, the whole study process has constituted a good opportunity to engage with national UHC actors on issues they had not reflected on before.

The study was also an opportunity for the CoPs to validate their own ability to undertake research alongside the participatory and inclusive values they promote. The study process and recommendations stemming from national workshops also confirmed that the new culture the CoPs are promoting at regional level is getting strong resonance at country level.

The enriching experience of this action-research is being shared within the CoPs. It has strengthened our conviction that there is space for more innovation in developing and leading knowledge agendas at the global, regional, sub-regional and country levels.

## Box 1 Examples of questions for each block

**Block 1: Leadership that reinforces learning**

The political level is aware that success of Universal Health Coverage (UHC) requires the mobilisation of many ministries, agencies and stakeholders, including non-public or international; it has set required dialogue platforms and mechanisms

The current leaders in my organisation support the priority given to UHC and adhere to the distribution of roles decided by the political level

**Block 2: Supportive learning environment**

In my organisation, personal development is a concern of supervisors – with their staff, they behave not as bosses but as coaches

In my organisation, the teams have enough flexibility to reorient their action on the basis of new information they have collected (e.g. following a field visit, information from the decentralised level)

The analytical contributions by Financial and Technical Partners are useful to inform progress towards UHC

**Block 3: Practical processes for learning**

In my organisation, the content of the learning agenda is fuelled by the needs identified by the individuals themselves or the deliberations and self-evaluations conducted at the team level

The leaders of my organisation recognise the importance of quantitative data to lead the country towards UHC

In our organisation, we have a mechanism to identify and promote good practices. We also identify mistakes and we make sure not to repeat them

## Box 2 Lessons learnt from the comparison

Each country could be singled out for some smart ideas on how to advance learning for Universal Health Coverage (UHC). Let us just flag a few. In Morocco, the creation of an inter-ministerial steering committee for UHC allowed a strategic positioning of the UHC agenda at the level of the government, with a clear benefit in reducing the gap between knowledge and strategic decision-making. Burkina Faso shows that even low-income countries can build strong learning ecosystems. Constant investment in health system research capacity pays off.

During our data collection, we realised the heuristic power of our framework: the process revealed some actors the different elements needed for a leadership supportive to systemic learning. The national workshops encouraged countries to take some concrete actions (with eventual variable success), for instance, by setting up knowledge platforms. Country reports were rich in recommendations.

The collective analysis of the study results in an international meeting showed that each country has something to share with others in terms of successes but also weaknesses. The dynamic we created throughout this research has triggered an exchange between countries as to how to better learn to achieve the UHC objectives.

## Abbreviations

CoPs: Communities of practice
LHS: Learning health system
LMICs: Low- and middle-income countries
LO: Learning organisation
LS: Learning system
UHC: Universal health coverage
